# Who understands and how? – exploring nurses’ perceptions of the readability and usability of instructions written by hospital pharmacists

**DOI:** 10.1186/s12913-025-12867-7

**Published:** 2025-05-17

**Authors:** Henri Satokangas, Kirsi Kvarnström, Hanna M. Tolonen

**Affiliations:** 1 Department of Finnish, Finno-Ugrian and Scandinavian Studies, Faculty of Arts, University of Helsinki, Unioninkatu 40, Helsinki, 00014 Finland; 2 HUS Pharmacy, HUS Helsinki University Hospital, Stenbäckinkatu 9, Helsinki, 00029 Finland; 3 Clinical Pharmacy Group, Division of Pharmacology and Pharmacotherapy, Faculty of Pharmacy, University of Helsinki, Helsinki, Finland

**Keywords:** Instruction, Usability, Readability, Hospital pharmacist, Communication, Medication management, Nurse

## Abstract

**Background:**

Clear and precise instructions are essential for high-quality medication management. This study focuses on identifying usability and functionality of instructional materials on medicine handling and use produced by hospital pharmacists. The secondary aim is to provide recommendations for hospital pharmacists on writing instructional texts.

**Methods:**

Semi-structured interviews were conducted with nurses who read and used medication-related instructions written by hospital pharmacists in a large tertiary hospital in Finland. Data were analyzed using inductive content analysis, incorporating an applied linguistic analysis of the grammatical and structural features of the Finnish-language instructional texts. The analysis focused on the nurses’ instruction-reading practices, identifying both problems and well-functioning dimensions of the texts, and proposing improvements for this type of communication between healthcare professionals.

**Results:**

The interviewed nurses (*n* = 9) consistently found the instructions somewhat challenging, primarily due to their length, complex structure and limited accessibility. While the interviewees stated that the instructions did not present personal difficulties, they expressed concern that other staff in the ward might struggle with the instructions. According to the interviews, readability problems can be mitigated using visual support. Linguistic features such as terminology and the interpretation of impersonal grammatical structures (e.g., passive voice) related to the division of labor were not seen as problematic for the interviewees. The usability of the instructions was enhanced by effective communication with the hospital pharmacists.

**Conclusion:**

When producing instructions for staff on the wards, it is important to focus on both internal text features, such as the use of graphics and consistent terminology, as well as external factors, like easy access communication between hospital pharmacists and ward staff.

## Background

The role of hospital pharmacists is to promote high-quality medication management, which includes supporting the rational use of medicines and ensuring that medicine supply complies with pharmaceutical legislation [1]. In Finland, while clinical pharmacy services in hospital wards have expanded [2], the responsibility for handling medicines often still lies with nurses. To ensure effective and safe medication practices, it is essential that each step of the medication process is clearly and thoroughly instructed. Finnish legislation mandates that hospital pharmacies are responsible for providing these instructions on the handling and use of medicines [3]. As a result, hospital pharmacists in Finland routinely develop detailed guidelines on medication management, primarily aimed at nurses and clinical pharmacists.

As these instructions are likely to influence the safe use of medicines, ensuring their readability and usability is essential. However, assessing and improving readability is complex, as automated readability tools often fail to capture how real readers perceive and interact with texts [4]. Functional approaches that view texts as communication within specific context provide more in-depth insights, especially when combined with feedback from actual users [5]. While qualitative methods have been used in pharmaceutical research, language-oriented perspectives are rare. Healthcare communication has traditionally been studied in applied linguistics, particularly instructional texts on medicine use [6–8]. These texts often seem more targeted toward doctors than patients despite the latter being their intended audience [7]. Additionally, research combining surveys and interviews shows that addressing the recipient directly, making the stages of action clear and specifying expected actions improve readability [5]. 

Hospital pharmacists and their instructions for other healthcare professionals are a novel subject in this field. Most studies on instructional texts about medicines focus on patient-oriented types of communication, such as research on medication package inserts, prescription labels, drug information leaflets, patient information leaflets, patient instructions for use, and dispensed (oral) medication instructions [9–13]. While some attention has been given to summaries of product characteristics aimed at healthcare professionals, research has primarily focused on accuracy and completeness, neglecting readability and textual organization [14–16]. Thus, the readability of instructions produced by professionals for professionals constitutes a research gap. This study assessed the usability and functionality of hospital pharmacy instructions among ward staff and provides recommendations for hospital pharmacists on developing instructional texts for the handling and use of medicines within hospitals.

## Methods

### Study context

In Finland, social and health care is organized into 21 wellbeing services counties [17]. The most demanding, specialized medical treatment is delivered through five university hospitals. Helsinki University Hospital (HUS) provides secondary and tertiary care to a population of 1.6 million in the capital region [18]. HUS operates in 23 hospitals and employs more than 27,000 professionals, the majority being nurses. Pharmaceutical and clinical pharmacy services at HUS and the wellbeing services counties of the region are provided by HUS Pharmacy, a unit of HUS. With over 500 employees, HUS Pharmacy is the largest hospital pharmacy in Finland.

Pharmacists at HUS Pharmacy provide hospital ward and outpatient clinic staff with general instructions on the management and use of medicines. Over the past decade, efforts have been made to standardize the structure of these guidelines, which are reviewed every 1–2 years to ensure they remain up to date. The content of the instructions addresses a wide range of topics related to medication, such as appropriateness reviews of hospital medication orders, tablet crushing, controlling and monitoring storage temperature, and reconstitution of medicines in clinical setting. These written guidelines serve as a critical communication tool between healthcare professionals and are designed to reach a broad and diverse audience. The body text of a typical instruction spans a few pages, often including one or more appendices. Thus, the length of the documents varies between 2 and 14 pages. At the time of the study, instructions were accessible to all HUS employees via the HUS Intranet.

### Study design and the interview guide

This qualitative study aimed to capture readers’ opinions on instructional texts through individual semi-structured interviews, the method suitable for previously unstudied topics [19]. This interview study was part of a larger project that examined Finnish-language instructions on the handling and use of medicines by considering the process, the product, and opinions – an approach previously reported in the linguistic literature [20]. The two additional perspectives addressed the process through group interviews with instruction writers (hospital pharmacists) and the product, i.e., the instructions, through a close linguistically oriented text analysis of instruction documents (*n* = 22) [21–23].

The reader interview guide (Table 1) was informed by a preceding textual analysis focusing on the lexico-grammatical (i.e., the interplay between vocabulary and grammar in a text), rhetorical, and structural features of the instruction documents (*n* = 22). The topics in the interview guide were motivated by the specific textual features observed in the text analysis such as structural elements and the morphosyntactic strategies. These strategies, used in directive language, refer to the grammatical and lexical choices that guide readers, such as the use of imperatives, modal verbs, and passive voice. For example, the previous analysis identified an abundant use of impersonal structures (i.e., passive voice and general third-person reference) and the practice of placing practical directions in appendices [23], which were then incorporated into the interview guide as discussion topics.

**Table 1:** The reader interview guide

1. Where are the instructions and how are they accessed? 2. In what kind of situations are the instructions read and who reads them? 3. Thinking aloud with an example text: what do you think while reading the instruction document? a) Is there something in the text that does not concern you? Do you skip passages? b) Is it laborious to read the instruction? c) Are there unclear passages, words or sentences? d) Is it clear who is being instructed in each place, for example, when passive verb form is used? 4. What kind of a reading experience are the instructions in general? 5. How clear is it who is being directed to perform each action and how the division of labor works? 6. Is it clear why the instructions have been written? 7. How would you improve the instructions so that they would better support your work?	

Three instruction documents written by hospital pharmacists were selected for review during the interviews. Instructions on tablet crushing, Instructions on medicine dispensing, and Instructions on medicine storage facilities were chosen to be the most relevant and represent diverse range of instructions. The assignment of a specific instruction text to each interview was done randomly by the interviewer.

### Participant recruitment and inclusion criteria

The inclusion criteria or the study were that participants (a) are nurses or have a background as nurses and (b) read regularly the instructions as part of their everyday work. Potential interviewees were recruited by convenience sampling from various medical specialties among HUS employees who regularly work with medicines and actively read the related instructions. The researchers initiated the recruitment process by sending an email to the quality managers of relevant clinical departments, requesting them to identify suitable candidates: nurse professionals who frequently consult these instructions. In addition, the study was promoted during hospital-based training sessions led by hospital pharmacists for healthcare professionals involved in medication management. The researcher assigned to conduct the interviews received contact details for 16 potential participants, either from quality managers or directly from the candidates themselves. Nine of these nurse professionals agreed to participate and provided informed consent. Interviews were scheduled (dates and times) with these professionals. The remaining seven candidates either did not respond to the interview invitation or failed to reply to proposals for rescheduling.

### Data collection

The interview guide was assessed for suitability and flow during the first interview. Since no modifications were needed, the first interview was included in the data. The semi-structured interviews were conducted by video meeting. Individual interviews were carried out by the first author (male with a background in applied linguistics) with previous experience in interviews. Only the interviewing researcher and the interviewee were present during the meeting, and they had not met beforehand. The video meetings were recorded, with recording starting after the interviewee gave oral consent. The interviewees were informed about the aim of the study (the instructional texts as the topic) as well as the use and storage of the interview data, whereafter written consent was obtained.

The interview guide was used flexibly, allowing the interviewer to ask follow-up questions, thus maintaining a conversational tone and encouraging interviewees to express the perspectives meaningful to them. Initially, everyday practices related to the instructions and the readability of the texts were addressed on a general level. Then, approximately midway through the interview, an instruction document was introduced to focus on linguistic and textual features. This part of the interview explored more specific elements of the texts, such as the use of passive form and general third-person reference [24], the use of imperative form, and the structure of the text. Both the interview and follow-up questions were informed by the prior text analysis, carefully phrased to avoid leading questions and encourage personal insights.

### Data analysis

The interview data were transcribed verbatim by the interviewer and analyzed using conventional inductive content analysis, a method well-suited for exploring topics without preexisting theories [25]. The main categories – issues, well-functioning practices, and suggestions for improvement – were identified through thematically-oriented close reading, coding, identification of subcategories, and interpretive discussion between the authors. The coding proceeded from categorizing observations into groups (e.g., different kinds of references to the length of the texts) to simplifications (e.g.,” length as a problem “) and furthermore to synthesis that resulted in the reported categories. The research team’s composition — one expert in applied linguistics and two with backgrounds in pharmacy — allowed for the integration of insights from diverse theoretical traditions, resulting in a more nuanced interpretation of the material [26]. In applied linguistics, a key approach to professional communication is to explore how readers perceive and make sense of communicative practices in their own words [27]. This aligns with the goals of qualitative social pharmacy research which seeks to capture health care practitioners’ views, beliefs, and perceptions [26]. In the results section, selected examples illustrate the broader ways interviewees addressed the topics. The study followed the COREQ checklist [28] for reporting where applicable.

## Results

Nine nurses or individuals with a nursing background who read instructions prepared by hospital pharmacists in their daily work participated in the interviews. All interviewees, comprising nurses and nurse managers, were native Finnish speakers. Each interview took between 17 and 30 min. As readers, the interviewees (*n* = 9) assessed the usability and functionality of the medication management instructions, identifying both ineffective aspects, i.e., obstacles for readability, and effective aspects, i.e., well-functioning practices that enhance the instructions’ readability and usability (Table 2). They also provided several suggestions for improvement.

**Table 2:** Obstacles for readability, well-functioning practices, and suggestions for improvement of the instructions written by hospital pharmacists, as perceived by interviewees (*n* = 9)

Obstacles for readability	• Length • Complex structure • Links and references in body text • Accessibility
Well-functioning practices	• Broker role • Terminology • Interpreting the division of labor • Specificity • Contacts with hospital pharmacists
Suggestions for improvement	• Concentrated instruction platform to improve accessibility • Clickable headings to ease navigation in lengthy documents • Visual support • Space for ward-specific instructions and exceptional situations

### Obstacles for readability of instructions

#### Length

**Fig. 1:**
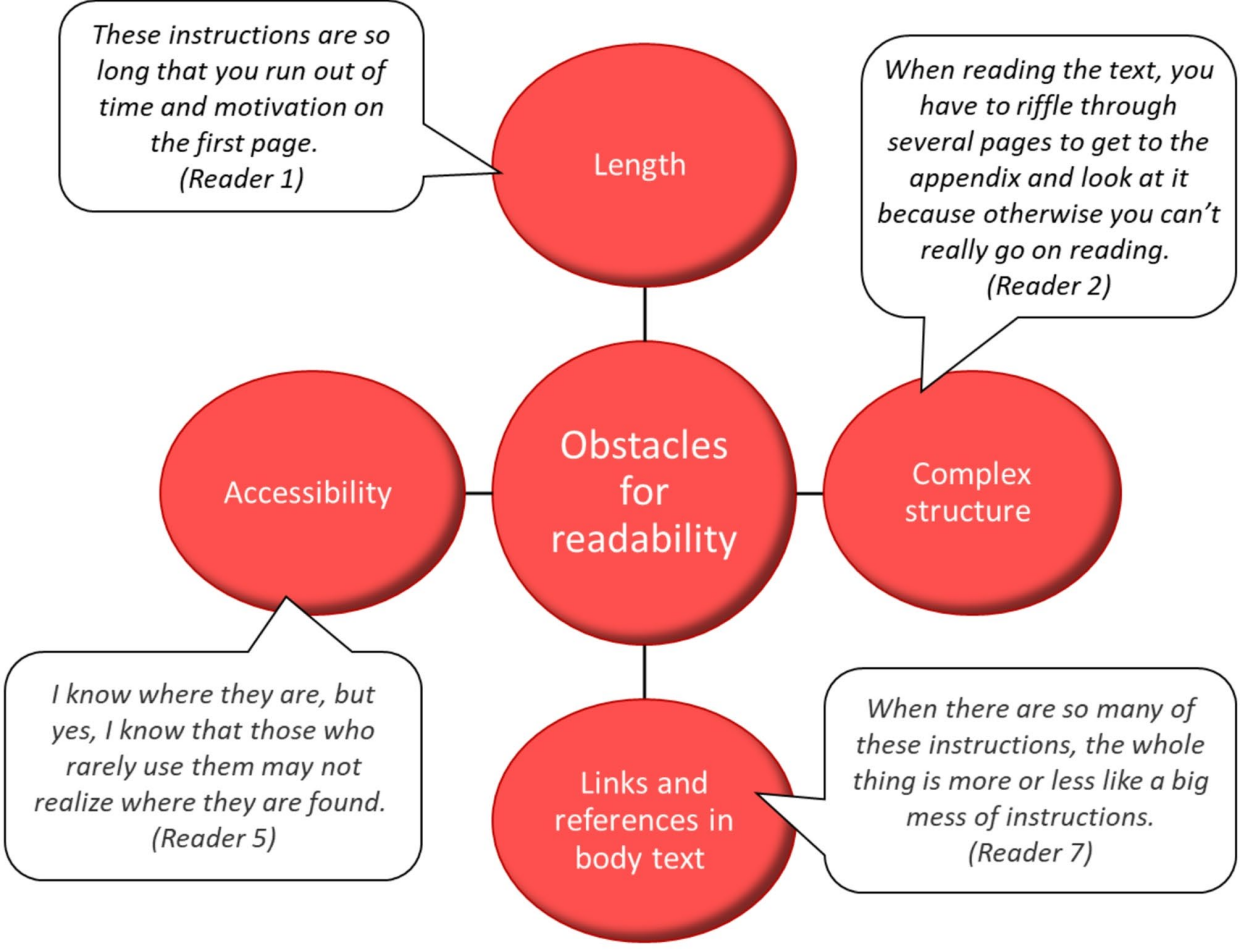
Obstacles for readability of the instructions as perceived by study participants (*n*=9)

The most frequently mentioned issue affecting the readability of the instructions was their length (Fig. 1). As a highly noticeable textual characteristic, length is an easy point of discussion when evaluating readability. However, the interviewees noted that omitting content is difficult since there is not really anything unnecessary included in them. Actual alternatives to the long instructions were considered limited. One participant suggested that some background information could be excluded, as it should already be covered in standard nursing education. Overall, the interviewees agreed that, upon review, all the information in the documents remained relevant.

#### Complex structure

Many interviewees described how reading and interpreting the instructions was initially challenging but became easier with experience. Appendices have an essential role in the instructions, often containing detailed, step-by-step procedures referenced in the main text. The readers describe the process of reading between the main text and appendix as “riffling” and “jumping” back and forth, which they find laborious (Fig. 1). After initially describing challenges with this format, one reader suggested an improvement: embedding the step-by-step procedures directly into the body text to create a more cohesive, unitary document. Despite the reported difficulties, the interviewees consistently noted that, with experience, they had adapted to the appendix-based structure and could navigate it fluently. Although the structural complexity posed challenges initially, especially for those unfamiliar with the format, it was ultimately not insurmountable once readers became accustomed to it.

#### Links and references in main text

Instructions on the handling and use of medicines are part of a broader network of instructional texts, with significant overlap between individual topics. As a result, explicit references and links play a central role in the instructions. The interviewees expressed mixed opinions about these textual elements: on one hand, references and direct links are practical, enabling smooth navigation through the interconnected network of instructions; on the other, long references, such as various references to legislation, and links within the main text can make it cluttered and difficult to read (Fig. 1).

#### Accessibility

The hospital’s intranet was deemed technically challenging to navigate, affecting the ability of ward staff to access the instructions (Fig. 1). Interestingly, the interviewees – who were regular users of the instructions – did not view accessibility as an issue for themselves but rather for other staff members. This pattern emerged consistently throughout the interviews: participants initially stated that they had no trouble locating the instructions due to their familiarity with the system, but then speculated that other staff likely struggled with this task.

### Well-functioning practices

#### Readers as brokers

**Fig. 2:**
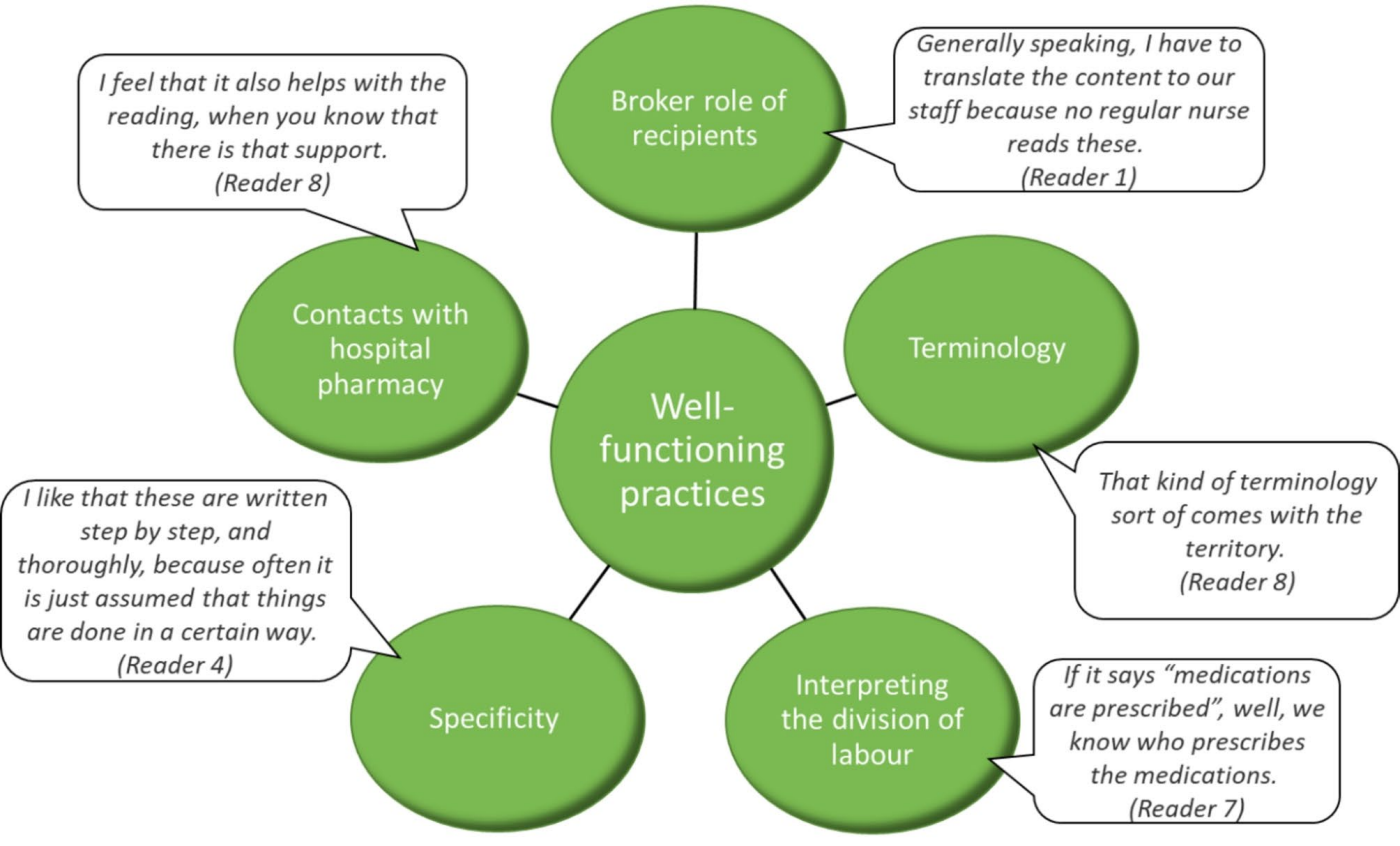
Instructions-related well-functioning practices identified by study participants (*n*=9)

According to the interviews (*n* = 9), familiarity with the instructions is largely confined to a specialized group of professionals, e.g., nurses responsible for medications, clinical pharmacists, and supervisors (Fig. 2). The interviewed nurses articulated in their discourse a role for themselves as brokers who read and interpret the instructions and convey the information to the rest of the ward staff. The interviewed individuals seemed generally satisfied with this role, as it is commonly recognized and other staff know who to approach when they need assistance.

However, one interviewee noted that this reliance on specialized readers might contribute to other staff not engaging with the instructions at all. This divergent reading practice ties into broader issues of accessibility, readability and document length. Many interviewees referred to a well-functioning division of labor, where specific individuals read and interpret the instructions, acting as intermediaries between the instructions and the rest of the ward staff. This practice was considered especially beneficial for employees still learning Finnish, as interviewees consistently felt the instructions were too complex for non-native speakers. These staff members were expected to overcome language challenges through collaboration and support from colleagues. From this perspective, it was considered unnecessary for everyone to read the instructions themselves, as specialized readers helped bridge both linguistic and practical gaps.

#### Terminology

The interviewees reported no difficulties with specialized terminology and consistently addressed it as a non-issue in terms of understandability. Specialized vocabulary was regarded as an inherent part of the medical field, something nurses are expected to be familiar with based on their professional education. If a particular term seemed unfamiliar, it was considered properly explained. Interestingly, some readers even commented that certain explanations of terms were unnecessary. The interviewees did, however, recognize that these terms might be difficult for others, though not for themselves. This mirrors a familiar pattern seen in discussions of accessibility and structural complexity: perceived difficulties were projected onto other staff members. Systematic use of terminology was highlighted as an important factor in readability. One interviewee noted that as part of the standardization of the instructions, the terminology had become more consistent, which further improved the clarity of the instructions.

#### Interpreting the division of labor

The instructions make extensive use of passive voice and general third-person reference to convey required actions. These grammatical choices emphasize the actions themselves while downplaying or omitting the specific agent responsible for the action. Despite this high level of impersonality, the interviewees did not find it problematic. Based on their everyday organizational knowledge, they are aware of who is responsible to perform the tasks outlined in the instructions.

#### Specificity

One interview question explicitly addressed the clarity and precision of the instructions. Generally, the responses were positive, with many praising the accuracy, that characterizes the instructions. One respondent even noted that the accuracy of expression had improved recently. This highlights the efforts toward standardization, particularly in the discussion on certain textual features (see also the terminological systematicity above). However, specificity is also flagged as a potential issue when ward-specific instructions are brought into focus (see below).

#### Effortless communication with hospital pharmacists beyond instructional texts

Instructional texts are only one of the many ways hospital pharmacists provide support and guidance on the handling and use of medicines. In the interviews, the nurses highlighted the significance of having easy access to communication with hospital pharmacists. One interviewee noted that simply knowing pharmacists’ support is readily available makes engaging with the instructional texts less burdensome.

### Suggestions for improvement

**Fig. 3:**
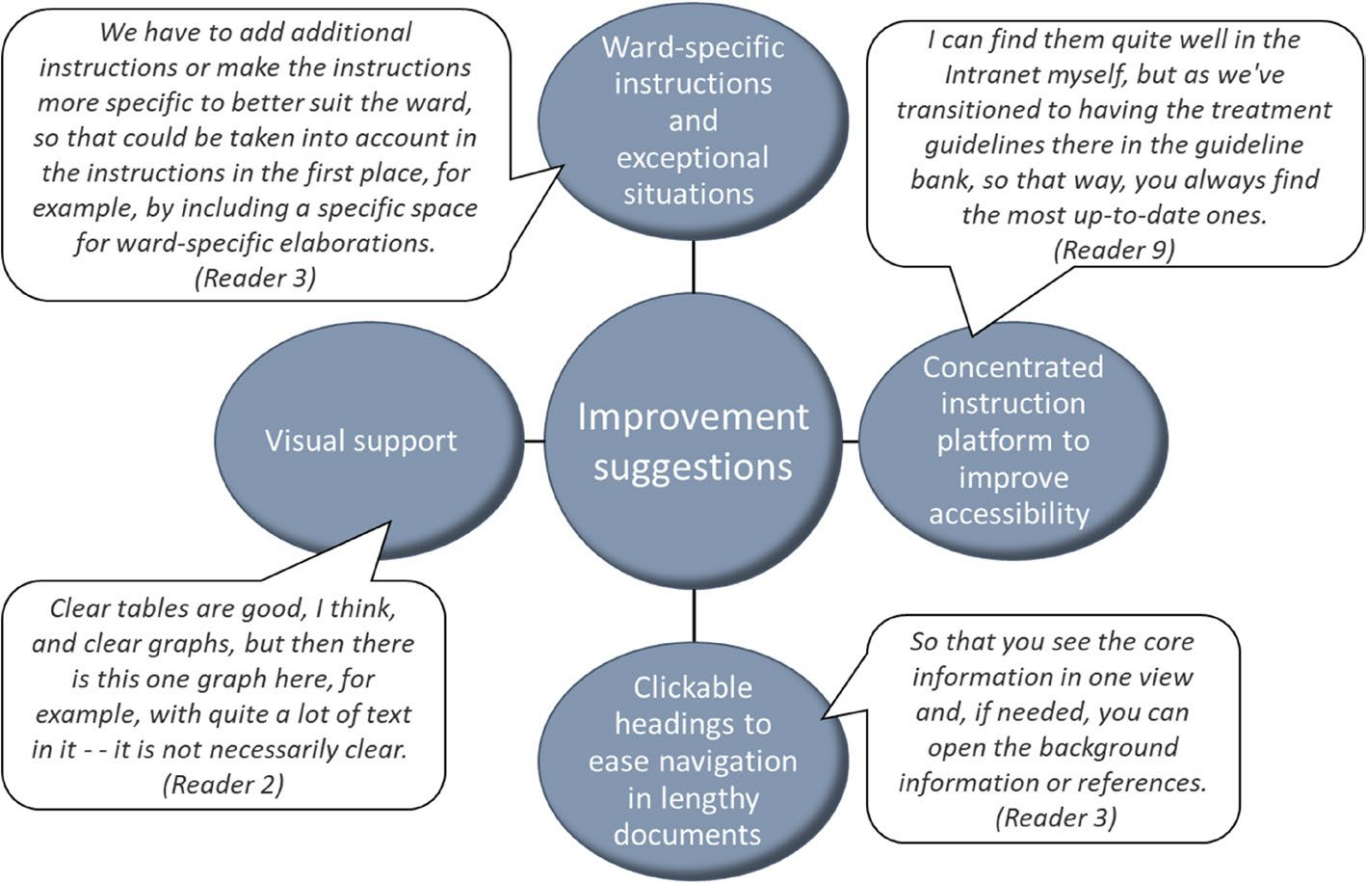
Study participants' (*n*=9) suggestions for improvement of instructions

The interviewees were invited to share their suggestions for improving the instructions. Some of these suggestions directly addressed the previously mentioned issues, such as accessibility and length, offering concrete solutions (Fig. 3). Other suggestions were more general, encouraging broader reflection on practical solutions.

#### Centralized instruction platform to improve accessibility

HUS maintains a centralized instruction platform, an “instruction bank,” where guidelines on various topics are consolidated. The interviewees were familiar with this platform and expressed a desire for it to include instructions on the handling of medicines.

#### Clickable headings to ease navigation in lengthy documents

One interviewee proposed a concrete technical solution: organizing the instructions under collapsible section headings that expand when clicked. This would allow optional subsections under clear, informative headings, making the content more easily understandable and enabling readers to navigate the text based on their specific needs. Currently, the instructions are presented as static PDF files, requiring the reader to view the entire document at once.

#### Visual support

Interviewees frequently mentioned the importance of visual tools, such as diagrams, flowcharts, checklists, tables, and use of colors, in supporting readability. This was a topic that was unanimously agreed upon: various visual elements make the instructions more approachable and easier to follow, particularly when reading in a hurry. However, attention must also be given to the design of the tables themselves.

Crucially, certain factors affecting readability, such as document length, can be alleviated through effective visual support. A well-structured layout with a table of contents and standardized subheadings can enhance clarity and usability. According to the interviewees, these visual aids can make the document easier to navigate and more adaptable, ultimately reducing the perception of reading the instructions as a burdensome task.

#### Space for ward-specific instructions and exceptional situations

As the instructions are intended for staff across a wide range of hospital wards, their audience consists of readers with varying responsibilities and backgrounds. Several interviewees highlighted the fact that each ward has its own specific needs. In practice, the instructions are adapted differently in daily routines across wards. One participant suggested that the instructions could benefit from greater specificity in outlining the roles and responsibilities of different professionals, such as clinical pharmacists and nurses. Additionally, one interviewee noted that the issue of excessive length could be addressed by omitting sections that are not relevant to all wards, such as ward-specific details on biological safety cabinets. 

## Discussion

This interview study with nurses who regularly use medicine-related instructions created by hospital pharmacists identified both effective and ineffective aspects of these instructions. Readability was hindered by factors such as excessive length, complex structure with appendices, links, and references, as well as perceived limited accessibility. While reading and interpreting the instructions were perceived as initially challenging, they became easier with experience. In contrast, features that improved readability included consistent terminology, a clear professional context with well-known responsibilities, and the accuracy of the instructions. Moreover, the participants reported their role as brokers between the instructions and other ward staff. They viewed their role in interpreting the instructions and their easy access to communication with hospital pharmacists as factors that improved the usability and understanding of the instructions.

Based on the findings of this study, some recommendations can be proposed for creating effective instructions from hospital pharmacists to ward staff. These can be grouped into three key areas (a) design and organization of the text, (b) technical accessibility, and (c) establishing easy access communication channels between ward staff and hospital pharmacists beyond the instructional documents. The applied linguistic approach to professional discursive practices typically emphasizes cooperation with practitioners and practical relevance [29]. This entails the aspiration to utilize the research findings to improve communicative practices in the organizations where the research takes place.

Firstly, different types of readers could be better accommodated through thoughtful structural solutions in the instructions. A more effective format could be bipartite: (1) a concise, straightforward summary of the procedures at the beginning, designed for all staff, followed by (2) a more detailed instructional section with in-depth background information, tailored for nurses responsible for medicines and clinical pharmacists. This structure would align with actual reading practices, where staff in broker roles tend to read the instructions thoroughly, while also encouraging wider readership by making the instructions more accessible to all staff members. By adopting this bipartite textual organization, the study’s findings on current reading habits would be integrated into the design, addressing issues related to length and structural complexity.

Several elements were identified in this study to support inexperienced readers and enhance the predictability and clarity of the text. Incorporating these elements can significantly improve the structure of instructional materials. Applying the findings of the interviews, practical suggestions can be made for future instruction documents:

- Concise table of contents
- Brief summary of procedures
- Explicit subheadings that appear in a similar order from one instruction to another
- Clearly framed references to legislation
- Images, bullet point checklists, spacious layout
- Consistent terminology

Visual and layout strategies function as tools for simplifying the textual organization [30]. By arranging and presenting textual elements thoughtfully, these strategies improve readability without sacrificing the precision or accuracy of the content.

Secondly, it is essential to ensure that instructions are easily accessible, enabling staff to locate them amidst the busy routines of the wards quickly. Interviewees highlighted the importance of integrating these instructions into the hospital’s general instruction platform, where they could be found alongside other critical guidelines. However, meeting the diverse needs of staff on individual wards is closely linked to the system’s technical capabilities. Interviewees suggested that customizable sections tailored to specific ward requirements or expandable, systematically organized headings could enhance usability. While they desired more precise, ward-specific guidelines, they acknowledged the complexity and challenges this would pose for the hospital pharmacists.

Thirdly, regarding communicative practices in healthcare organizations, it is noteworthy that the nurses emphasized the strong link between understanding written instructions and maintaining effective communication with hospital pharmacists. Open, easy access communication – such as familiar pharmacists and direct contacts to hospital pharmacy staff – was considered essential by the interviewees. Indeed, the interviews indicated that instructions are not standalone documents but part of a broader network of communication practices between the staff at the hospital pharmacy and those on the wards.

The study reveals notable differences between this type of communication used among healthcare professionals and previously examined instructional texts intended for patients [4, 5]. First, the nurses interviewed did not encounter difficulties in interpreting impersonal grammatical structures, which are often challenging for patients. Second, while specialized vocabulary is frequently identified as a major obstacle to readability for patients, the nurses in this study did not find the terminology problematic. These contrasting results underscore the need to study communication practices among healthcare professionals in a way that is sensitive to specific context and tailored to the need of different professionals. For example, communication practices and needs may differ in the operating room compared to primary healthcare, and understanding these differences can lead to more effective communication within different healthcare settings.

Formally, the instructions written by hospital pharmacists are intended for all hospital staff in wards and other units. In our previous study, hospital pharmacists explicitly express this broader target audience in their interviews [22]. However, since the interviewed readers perceived themselves as brokers, it’s important for hospital pharmacists to consider the difference between imagined and actual readership: who are the instructions truly aimed at, and whose reading experience should be prioritized? Based on this study, no specific recommendations can be made; rather, further research is needed to explore this apparent contradiction.

The participants in this study were staff members who identified themselves as brokers, facilitating communication between the written instructions and other ward staff. Due to challenges in participant recruitment, the number of interviewees was limited, and all volunteers were included in the study. Despite this significant limitation, the data saturation was reached, as no new themes or subcategories emerged in the last interviews. A notable limitation is that all the interviewed nurses were already actively engaged with these instructions as part of their daily work, which was likely influenced by the convenience sampling approach. This limits the transferability and generalizability of the study’s findings. In addition, it should be noted that healthcare systems are organized differently across countries, which should be considered when applying these findings to other settings.

Future research should focus on the readability of such materials among staff who do not routinely interact with them, particularly those with Finnish as a second language. Several interviewees mentioned both imagined and reported difficulties in understanding the instructions among non-Finnish-speaking staff. In practice, however, professional language is often acquired during everyday tasks in multilingual work environments [31], providing a compelling context for further study. As with any qualitative research, the potential for researcher bias exists. To address this, the research team engaged in ongoing discussions throughout the analysis phase to ensure balanced and accurate interpretations. Despite its limitations, this study represents a novel contribution to understanding communication practices among healthcare professionals.

## Conclusion

Internal text features, such as graphics and systematic use of terminology, technical solutions, and easy access communication practices between hospital pharmacists and ward staff play essential roles in the readability of medication instructions. Enhancing these features can improve the clarity and usability of these instructions for ward staff, ultimately contributing to the safety of the handling and use of medicines within hospitals.

## Data Availability

Original data are in Finnish. All requests should be directed to the corresponding author.
